# Transmission of Influenza Virus in a Mammalian Host Is Increased by PB2 Amino Acids 627K or 627E/701N

**DOI:** 10.1371/journal.ppat.1000252

**Published:** 2009-01-02

**Authors:** John Steel, Anice C. Lowen, Samira Mubareka, Peter Palese

**Affiliations:** 1 Department of Microbiology, Mount Sinai School of Medicine, New York, New York, United States of America; 2 Department of Medicine, Mount Sinai School of Medicine, New York, New York, United States of America; University of North Carolina, United States of America

## Abstract

Since 2003, more than 380 cases of H5N1 influenza virus infection of humans have been reported. Although the resultant disease in these cases was often severe or fatal, transmission of avian influenza viruses between humans is rare. The precise nature of the barrier blocking human-to-human spread is unknown. It is clear, however, that efficient human-to-human transmission of an antigenically novel influenza virus would result in a pandemic. Influenza viruses with changes at amino acids 627 or 701 of the PB2 protein have been isolated from human cases of highly pathogenic H5 and H7 avian influenza. Herein, we have used the guinea pig model to test the contributions of PB2 627 and 701 to mammalian transmission. To this end, viruses carrying mutations at these positions were generated in the A/Panama/2007/99 (H3N2) and A/Viet Nam/1203/04 (H5N1) backgrounds. In the context of either rPan99 or rVN1203, mutation of lysine 627 to the avian consensus residue glutamic acid was found to decrease transmission. Introduction of an asparagine at position 701, in conjunction with the K627E mutation, resulted in a phenotype more similar to that of the parental strains, suggesting that this residue can compensate for the lack of 627K in terms of increasing transmission in mammals. Thus, our data show that PB2 amino acids 627 and 701 are determinants of mammalian inter-host transmission in diverse virus backgrounds.

## Author Summary

To cause a pandemic, an influenza virus must transmit efficiently from human to human. The viral factors that enable person-to-person spread of influenza viruses remain elusive. Using the guinea pig, an animal which we have previously shown to model the human transmission of influenza, we have identified two specific residues in the viral polymerase, at PB2 positions 627 and 701, that can contribute to efficient transmission. Interestingly, the two adaptive mutations examined act independently to achieve the same phenotype. Furthermore, these residues impact the transmission of both H3N2 and H5N1 subtype influenza viruses in the context of a mammalian host. The common importance of these amino acids to two diverse virus strains—the human-adapted H3N2 and the more avian-like H5N1—indicates that their mutation may be a common route to the development of a transmission-competent virus. These findings suggest one feature that contributes to the making of a pandemic influenza virus.

## Introduction

Repeated introductions of avian H5N1 influenza viruses into the human population have resulted in more than 380 reported cases of severe disease since 2003. Greater than half of these cases have been fatal, highlighting the extreme pathogenicity of H5N1 influenza in humans. Furthermore, high viral loads have been detected in clinical specimens collected from infected patients [1]. Nevertheless, H5N1 influenza viruses do not transmit efficiently from person-to-person. Prior to the 1997 and ongoing 2003 outbreaks of H5N1 zoonoses, it was generally assumed that an influenza virus with the traits of efficient viral growth and pathogenicity in a given host species would transmit between individuals of that species. The phenotype of H5N1 viruses in humans has changed that view: it is now clear that specific viral factors are required to support inter-host transmission. The consequences of human-to-human spread of H5N1 influenza viruses would be of pandemic proportions; for this reason, significant effort has been expended in recent years towards the identification of molecular determinants of transmission. As a result of these efforts, it has become clear that transmissibility among humans is a complex and polygenic trait [2],[3]. Affinity of the viral hemagglutinin (HA) protein for α-2,6 linked sialic acid residues has been shown to be necessary [4] but not sufficient [2],[5] to support transmission between ferrets. Due to its importance in determining viral host range and pathogenicity, the viral polymerase complex has also been suggested to play a role in transmission [3],[6], but this hypothesis has not been tested directly. Additional viral proteins as well as host and environmental factors [7],[8] are also likely to impact the success of human-to-human spread of influenza viruses.

Amino acid 627 of the PB2 protein is almost exclusively a lysine in human influenza isolates and a glutamic acid in avian influenza isolates. This residue was first recognized as a determinant of host range in 1993 [9], and was later shown to contribute to the temperature sensitivity of avian viral replication in mammalian cells [10]. In particular, the introduction of a lysine at position 627 of an avian virus PB2 protein greatly improved replication of a minigenome segment in cells incubated at 33°C [10]. This finding has also been confirmed in the context of infected cell cultures: the growth of A/Viet Nam/1204/04 virus (VN1204; H5N1) in mammalian cell lines is improved at 33°C, but not 37°C or 41°C, by the single amino acid change, PB2 E627K. Furthermore, rVN1204 possessing 627K was found to grow more efficiently in the nasal turbinates of mice than the wild-type virus [6]. A recently reported observation provides some insight into the mechanism underlying the effects of the E to K change: the introduction of E at 627 into the WSN PB2 appears to decrease its association with the NP protein, in mammalian but not avian cells [11]. In addition to affecting host range and tissue tropism, PB2 627K has also been identified as a major pathogenicity determinant of H5N1 and H7N7 subtype influenza viruses in mammalian hosts [12]–[15]. Perhaps the most striking aspect of the polymorphism at PB2 627 is the rapidity with which lysine at this position arises when an avian influenza virus infects either a mouse or a human. A single passage in mice has been reported to be sufficient for an E627K variant to dominate the resultant H5N1 virus population [6],[16]. The presence of PB2 627K has furthermore been identified in multiple human isolates of H5N1 viruses [1],[17],[18].

Similarly to E627K, a change of PB2 amino acid 701 from aspartic acid to asparagine has been implicated in expanding the host range of avian (or avian-like) H5N1 and H7N7 subtype viruses to include mice [19],[20] and humans [1]. In addition, an asparagine at PB2 701 is a common feature of avian-like H3N2 swine viruses circulating in Europe. A mechanism has recently been proposed to explain the contribution of D701N to improved growth of a mouse adapted avian-like H7N7 virus, SC35M, in mammalian cell culture: D701N appears to enhance the binding of PB2 to importin α1 and correspondingly increase PB2 levels in the nucleus in mammalian, but not avian, cells [21].

A potential correlation between the polymorphisms at PB2 positions 627 and 701 was observed in H5N1 viruses collected from humans in 2004–2005. de Jong et al. reported that, among twelve clinical isolates, eight possessed 627K while a distinct three viruses carried 701N. The authors suggested that 701N may compensate for the lack of 627K in the context of mammalian cells [1].

Herein, we have evaluated two hypotheses: i) PB2 627K promotes the transmission of influenza viruses between mammals through improved growth in the upper respiratory tract, and ii) an asparagine at PB2 701 can functionally replace 627K in supporting viral growth and transmission in mammals. We used the guinea pig model to test the effects of polymorphisms at PB2 positions 627 and 701 on aerosol transmission of the recombinant human H3N2 isolate, A/Panama/2007/99 (rPan99), which we have previously shown to transmit with high efficiency [22], and on contact transmission of the recombinant human H5N1 isolate, A/Viet Nam/1203/04 (rVN1203). We show that rVN1203 transmits with moderate efficiency by the contact route in the guinea pig model. Furthermore, the mutation K627E decreases the transmission efficiency of both rPan99 and rVN1203 viruses. Contrary to previous reports and to our first hypothesis, we did not, however, observe a marked difference between the 627K and 627E containing viruses in terms of peak viral titers attained *in vivo*. Our second hypothesis did prove correct in that combination of glutamic acid at 627 with an aspartic acid to asparagine change at position 701 rescues the phenotype of the 627E viruses: rPan99 virus with 627E+701N transmits with similar efficiency to the parental rPan99 virus, while the rVN1203 627E 701N virus transmits with higher efficiency than the wild-type strain. Our data show that specific adaptations of the viral polymerase to the mammalian host support the transmission of influenza viruses of diverse lineage, revealing one viral trait that could contribute to the making of a pandemic strain.

## Results

### Rescue and in vitro characterization of recombinant viruses

Reverse genetics systems for Pan99 and VN1203 viruses were used to generate recombinant viruses which encode the mutations K627E alone or K627E and D701N combined, in their respective PB2 segments. All four mutant viruses and the two recombinant parental viruses were successfully recovered from cDNA transfection. Reverse transcription followed by PCR and sequencing of the PB2 gene segment of each virus confirmed that the expected sequence was present. As sequencing was only performed on the PB2 segment, it cannot be formally excluded that random mutation of the remaining segments may have contributed to the phenotypes observed. The PB2 genotype of each virus is summarized in Table 1.

**Table 1:** Amino acids at PB2 positions 627 and 701 of viruses used in this study.

	Residue 627 of PB2	Residue 701 of PB2
rPan99 wild type	K	D
rPan99 627E	E	D
rPan99 627E 701N	E	N
rVN1203 wild type	K	D
rVN1203 627E	E	D
rVN1203 627E 701N	E	N

To confirm that the three rVN1203 viruses possessed the polybasic cleavage site associated with high pathogenicity in H5N1 influenza viruses, growth in the absence of exogenous trypsin was assessed by plaque assay on MDCK cells. None of the viruses required trypsin for plaque formation, in contrast to the laboratory-adapted strain A/Puerto Rico/8/34 (data not shown).

**Figure 1:**
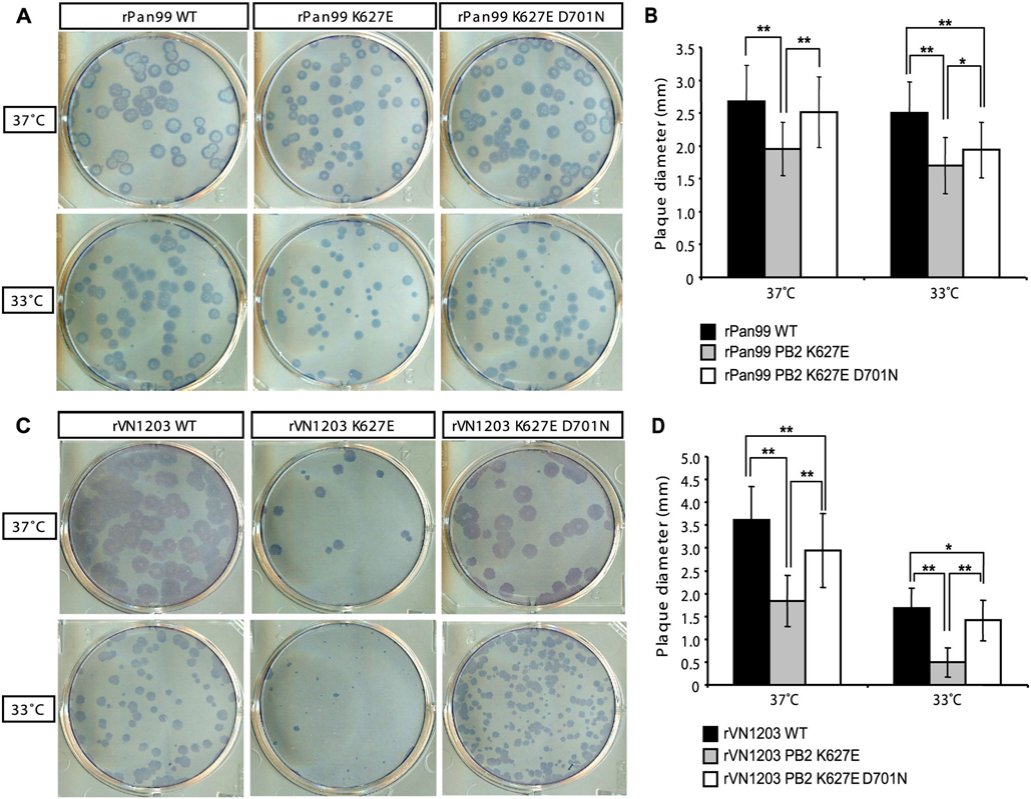
Plaque phenotypes of wild-type, PB2 627E and PB2 627E 701N viruses at 33°C and 37°C. Plaque assays were performed on MDCK cells incubated at 33°C or 37°C, as indicated at the left of the figure, and immunostained with virus-specific antibody. (A) Plaque phenotypes of rPan99 WT and PB2 mutant viruses. (B) Average plaque diameter for each rPan99 virus is plotted at 33°C and 37°C. (C) Plaque phenotypes of rVN1203 WT and PB2 mutant viruses. (D) Average plaque diameter for each rVN1203 virus is plotted at 33°C and 37°C. Plaque assays of rPan99 viruses and rVN1203 viruses were performed at different times. Error bars indicate standard deviation. * indicates p<0.01 and ** indicates p<0.001, as determined by t-test of plaque diameter values.

A preliminary estimate of the fitness of each recombinant virus was obtained by evaluating their plaque phenotypes on MDCK cells. Since previous reports indicate that the polymorphism at PB2 627 impacts viral growth at decreased temperature, plaque assays were performed in duplicate and incubated at either 37°C or 33°C. As seen in Figure 1A and 1B, at both 37°C and 33°C, rPan99 produced the largest plaques of the rPan99 derived viruses. The rPan99 PB2 627E virus formed slightly smaller plaques than either wild-type or rPan99 627E 701N viruses (p<0.001 at 37°C; p<0.001 relative to wild-type at 33°C, and p<0.01 relative to rPan99 627E 701N at 33°C). The rPan99 virus encoding PB2 627E 701N produced plaques which were not significantly smaller than rPan99 wild-type at 37°C (p>0.05) and plaques which were slightly smaller than the wild-type at 33°C (p<0.001, Figure 1A and 1B).

As was seen with the rPan99 series of viruses, the wild-type rVN1203 (PB2 627K) virus produced larger plaques than either the 627E or the 627E 701N mutant viruses (Figure 1C and 1D). The plaque size of all three viruses was reduced at 33°C compared to 37°C, but the reduction was most striking for the rVN1203 virus encoding PB2 627E. This virus produced pinpoint plaques at 33°C, indicating a significant impairment in plaque formation at the lower temperature. At 37°C the average diameter of plaques formed by rVN1203 627E was approximately 2.0-fold smaller than the wild-type (p<0.001); while at 33°C the plaques of rVN1203 627E were approximately 3.4-fold smaller than rVN1203 wild-type (p<0.001). Thus, rVN1203 627E was attenuated under both growth conditions, but attenuation was greater at 33°C. Similar to the rPan99 viruses, the rVN1203 virus encoding PB2 627E 701N produced plaques which were closer in size to the rVN1203 wild-type virus (1.2-fold reduced at 33°C, p<0.01, and 1.2-fold reduced at 37°C, p<0.001; Figure 1C and 1D).

### Multicycle growth of recombinant wild-type and PB2 mutant viruses in MDCK cells

**Figure 2:**
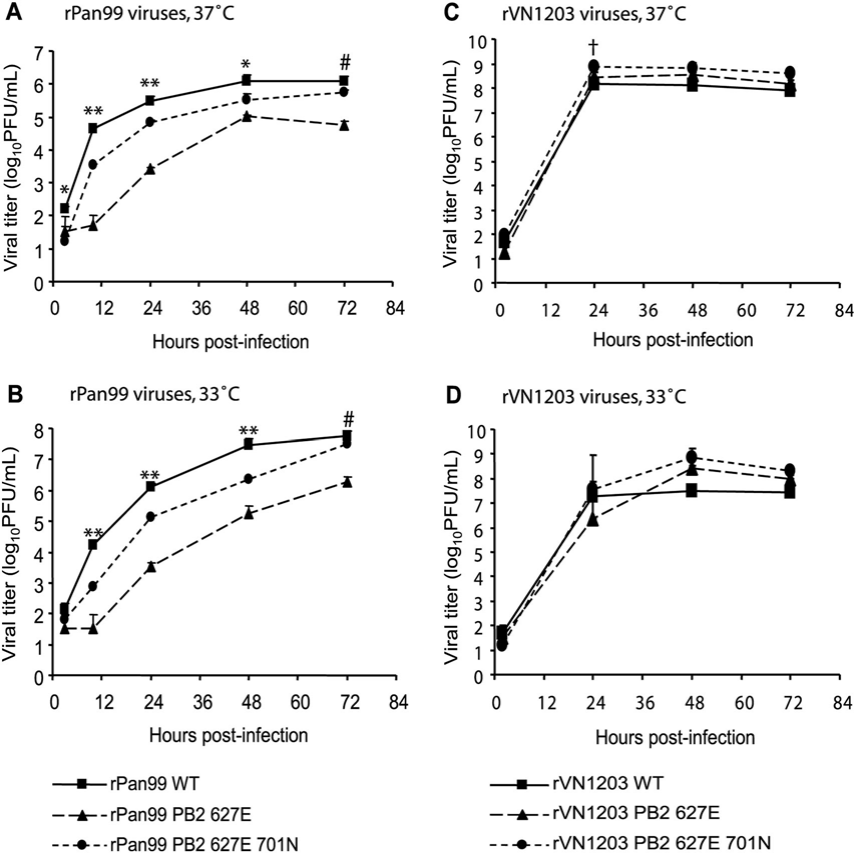
Growth kinetics of wild-type, PB2 627E and PB2 627E 701N viruses in MDCK cells incubated at 37°C and 33°C. (A) and (B) show the results from rPan99-based viruses, while (C) and (D) show the results from rVN1203-based viruses. Titers of the wild-type viruses are shown with squares and solid lines; titers of PB2 627E mutants are shown with triangles and long dashed lines; and titers of PB2 627E 701N viruses are shown with circles and short dashed lines. The MOI of infection in all cases was 0.01 PFU/cell. ** all three viruses are significantly different from each other (p<0.05, Student's t-test). * the titers of both mutants are significantly different from those of the wild-type (p<0.05, Student's t-test). # the titers of the 627E mutant are significantly different from those of both the wild-type and the 627E 701N viruses. † the titers of the 627E 701N double mutant are significantly different from those of both the wild-type and the 627E single mutant viruses.

To test whether the PB2 627E mutation results in a cold sensitive growth phenotype in the Pan99 background, the multi-cycle growth of the rPan99 based viruses in MDCK cells was compared at 33°C and 37°C. Cells were inoculated at low multiplicity (0.01 PFU/cell) and supernatant was sampled at 3, 10, 24, 48, and 72 h p.i. As shown in Figure 2, all three rPan99 based viruses grew to higher titers when incubated at 33°C than at 37°C. This result probably reflects the fact that Pan99 virus is highly adapted to growth in the upper respiratory tract of humans. At either temperature, the wild-type virus was found to yield the highest titers. The PB2 627E mutant was attenuated relative to the wild-type virus by greater than 10-fold at both 37°C and 33°C and at all time points except the first. Thus, in the Pan99 background, PB2 627E is an attenuating mutation, but does not appear to result in cold-sensitivity as seen in avian influenza viruses [6]. This lack of cold-sensitivity is most likely due to the presence of multiple adaptations to low temperature growth in the context of a human-adapted virus. At 33°C and 37°C, the rPan99 627E 701N virus grew to intermediate titers relative to the rPan99 and rPan99 627E viruses. These results suggest that, in MDCK cell culture, the attenuated phenotype of a K627E mutant is partially rescued by the D701N mutation.

A similar experiment was then performed with the rVN1203-based viruses. MDCK cells were inoculated at an MOI of 0.01 with rVN1203, rVN1203 627E or rVN1203 627E 701N viruses and supernatant was sampled at 2, 24, 48, and 72 h.p.i. As shown in Figure 2, there were no marked differences in the viral yields attained at either temperature. All three viruses grew to high titers by 24 h.p.i., although, at 33°C, the 627E mutant virus grew to 8.5-fold lower titers than the wild-type.

### Growth of rPan99, rPan99 627E, and rPan99 627E 701N in the upper and lower respiratory tracts of guinea pigs

**Figure 3:**
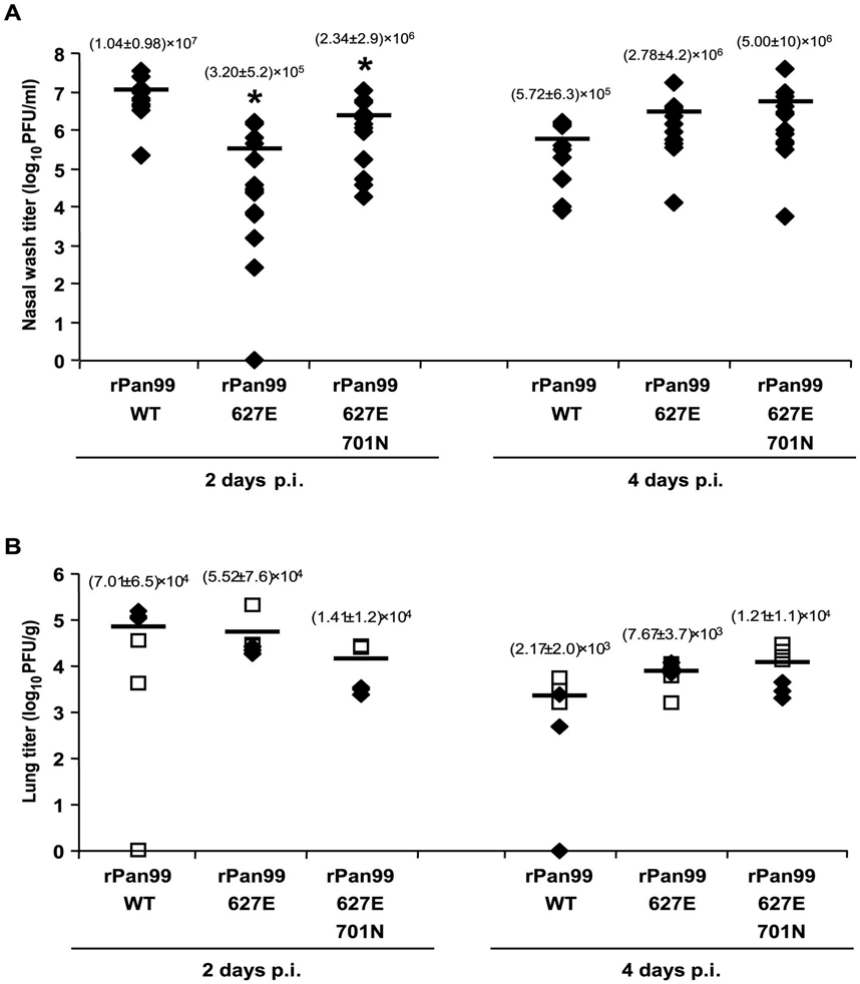
Growth kinetics of rPan99 wild-type, rPan99 627E and rPan99 627E 701N viruses in the upper and lower respiratory tracts of guinea pigs. (A) Viral titers in nasal wash samples of infected guinea pigs are plotted on days 2 and 4 p.i. On day 2 p.i., titers of the wild-type virus were significantly different from rPan99 627E (p = 0.0020) and from rPan99 627E 701N (p = 0.0057). On day 4 p.i., titers of the wild-type virus were not significantly different from those of either PB2 mutant virus (p≥0.03). (B) Titers of virus recovered from the supernatant of homogenized lung tissue at 2 d and 4 d p.i. Virus in three tissue samples from each of two animals was quantified; titers from one guinea pig are shown with open squares and titers from the second guinea pig are shown with closed diamonds. Titers of the wild-type virus were not significantly different from rPan99 627E (p = 0.39 on day 2 and p = 0.011 on day 4) or from rPan99 627E 701N (p = 0.067 on day 2 and p = 0.025 on day 4). All p-values were determined by Student's t-test. * indicates a significant difference relative to the wild-type, with p<0.01 as determined by Student's t-test. The mean and standard deviation for each data set are shown above the corresponding data points.

To test whether the phenotypes of rPan99 627E and rPan99 627E 701N seen *in vitro* are also manifested *in vivo*, we evaluated the titers of these viruses in the nasal passages and lungs of infected guinea pigs. Growth in the upper respiratory tract was assessed by collecting nasal lavage from intranasally inoculated animals on days 2 and 4 p.i. As shown in Figure 3A, rPan99 wild-type virus grew to a titer of 10^7^ PFU/mL at 2 d p.i. At this time point, the rPan99 627E virus produced a 30-fold lower titer. The kinetics of growth of the 627E mutant virus were delayed relative to the wild-type, with a higher titer (of 2.3×10^6^ PFU/mL) being reached at 4 d than at 2 d p.i. The rPan99 627E 701N double mutant exhibited an intermediate phenotype: titers on day 2 p.i. were only 4-fold lower (as compared to the 30-fold reduction for rPan99 627E) relative to rPan99 wild-type. Thus, moderate differences in viral titers achieved in the upper respiratory tract were observed between the three Pan99-based viruses, but this difference was observed only at day 2, and not day 4, post-inoculation.

Growth in the lower respiratory tract was assessed by titrating virus harvested from homogenates of guinea pig lung collected on days 2 and 4 p.i. Growth in the lungs was affected to a lesser extent by the K627E and D701N mutations than growth in the nasal passages. Clearance of the wild-type virus from the lungs appeared to be initiated more quickly than for the two mutant viruses; nevertheless, average titers of all three viruses were comparable on day 2 p.i. (2.4–7×10^4^ PFU/g) and day 4 p.i. (2.2–12×10^4^ PFU/g) (Figure 3B).

### Aerosol transmission of rPan99, rPan99 627E, and rPan99 627E 701N

**Figure 4:**
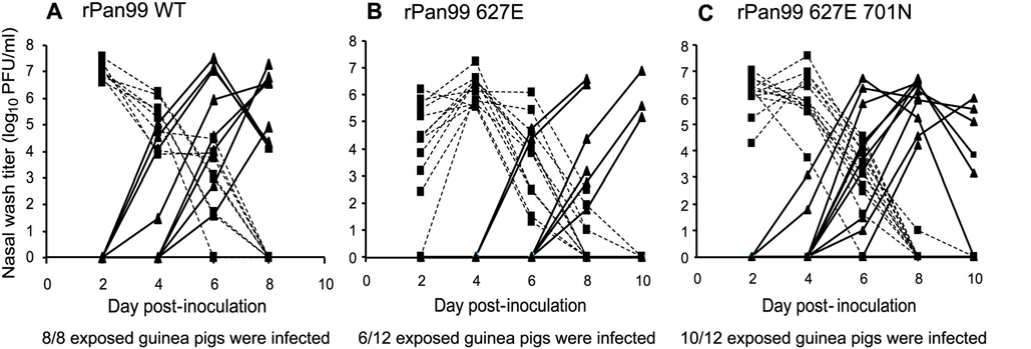
Aerosol transmission of rPan99 wild-type, rPan99 627E and rPan99 627E 701N viruses in guinea pigs. Nasal wash titers are plotted as a function of time post-inoculation. Titers of intranasally inoculated animals are represented by dashed lines and filled squares; titers of exposed guinea pigs are shown with solid lines and filled triangles. (A) The cumulative results of two experiments with rPan99 wild-type virus are shown. All four exposed guinea pigs were infected in both experiments. (B) The cumulative results of three experiments with rPan99 627E are shown. One of four, three of four and two of four exposed guinea pigs became infected in the first, second and third experiments, respectively. (C) The cumulative data from three experiments with rPan99 627E 701N are shown. Two of four, four of four, and four of four exposed guinea pigs became infected in the first, second, and third experiments, respectively.

We used the guinea pig model to characterize the transmission phenotypes of rPan99 wild-type, rPan99 627E and rPan99 627E 701N viruses. Two (rPan99 wild-type) or three (rPan99 627E and rPan99 627E 701N) independent experiments were performed with each virus, in which we assessed the rate of spread by the aerosol (large or small respiratory droplet) route. Each experiment involved eight guinea pigs: four infected intranasally with 1000 PFU of the appropriate virus, and four exposed animals. The rPan99 wild-type virus transmitted to all eight exposed guinea pigs (Figure 4A), as expected based on previous results with the non-recombinant Pan99 virus [7],[22]. The PB2 mutant virus, rPan99 627E, transmitted less efficiently, with just six of twelve exposed animals contracting infection, and with slower kinetics relative to the wild-type (Figure 4B). Partial rescue of the transmission defect was observed when the PB2 D701N change was also present: rPan99 627E 701N virus transmitted to ten of twelve exposed guinea pigs (Figure 4C). Transmission to contact animals, as measured by isolation of virus in nasal washings, was verified by hemagglutination inhibition assay of paired sera for each guinea pig (Table 2). To confirm that the mutant viruses had not reverted to the wild-type PB2 sequence prior to transmission, the PB2 genes of isolates from two exposed guinea pigs were sequenced for each virus. All transmitted viruses examined retained the introduced mutations. Thus, in the background of a human H3N2 isolate, mutation of PB2 residue 627 from K to E decreases the efficiency of transmission, while the introduction of D701N alongside K627E results in a phenotype more similar to that of the wild-type virus.

**Table 2:** Virus detection and seroconversion in guinea pigs inoculated or exposed as part of rPan99 virus transmission experiments.

		rPan99 wild-type		rPan99 PB2 627E		rPan99 PB2 627E 701N	
		Virus detection*	Seroconversion**	Virus detection*	Seroconversion**	Virus detection*	Seroconversion**
Expt. 1	Inoculated 1	+	+	+	+	+	+
	Inoculated 2	+	+	+	+	+	+
	Inoculated 3	+	+	+	+	+	+
	Inoculated 4	+	+	+	+	+	+
	Exposed 1	+	+	−	−	+	+
	Exposed 2	+	+	−	−	+	+
	Exposed 3	+	+	+	+	+	+
	Exposed 4	+	+	+	+	+	+
Expt. 2	Inoculated 1	+	+	+	+	+	+
	Inoculated 2	+	+	+	+	+	+
	Inoculated 3	+	+	+	+	+	+
	Inoculated 4	+	+	+	+	+	+
	Exposed 1	+	+	+	+	+	+
	Exposed 2	+	+	+	+	+	+
	Exposed 3	+	+	+	+	+	+
	Exposed 4	+	+	−	−	+	+
Expt. 3	Inoculated 1	n/a#	n/a	+	+	+	+
	Inoculated 2	n/a	n/a	+	+	+	+
	Inoculated 3	n/a	n/a	+	+	+	+
	Inoculated 4	n/a	n/a	+	+	+	+
	Exposed 1	n/a	n/a	−	−	+	+
	Exposed 2	n/a	n/a	−	−	−	−
	Exposed 3	n/a	n/a	+	+	+	+
	Exposed 4	n/a	n/a	−	−	−	−

*: limit of detection was 10 PFU/ml.

**: seroconversion was defined as a ≥4-fold increase in hemagglutination-inhibition titer.

# n/a not applicable; a third experiment was not performed with rPan99 wild-type.

### Contact transmission of rVN1203, rVN1203 627E, and rVN1203 627E 701N

**Figure 5:**
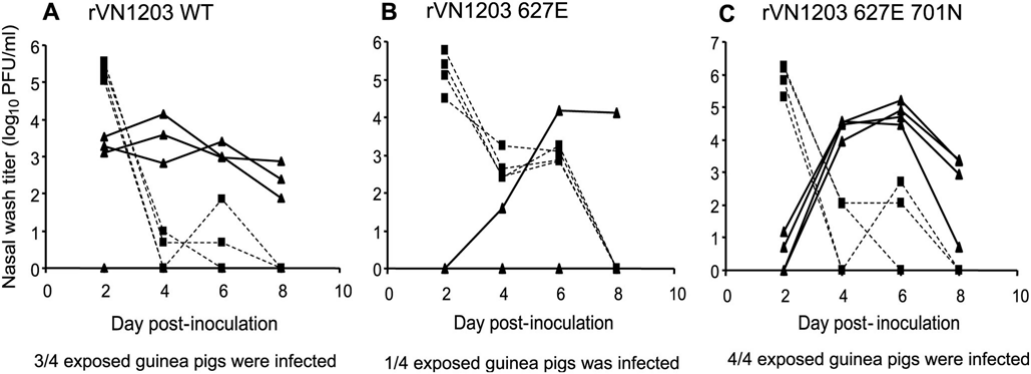
Contact transmission of rVN1203 wild-type, rVN1203 627E and rVN1203 627E 701N viruses in guinea pigs. Nasal wash titers are plotted as a function of time post-inoculation. Titers of intranasally inoculated animals are represented by dashed lines and filled squares; titers of exposed guinea pigs are shown with solid lines and filled triangles. (A) Transmission of rVN1203 wild-type virus. (B) Transmission of the rVN1203 627E mutant. (C) Transmission of the rVN1203 627E 701N double mutant.

To test whether the introduction of K627E or K627E D701N mutations would also alter the transmissibility of the rVN1203 virus, contact transmission experiments were performed in guinea pigs. Each experiment involved eight guinea pigs: four infected intranasally with 10^4^ PFU of the appropriate virus, and four exposed animals. In this case, each exposed guinea pig was placed in direct contact with an inoculated animal by placing them in the same cage at 24 h p.i. It is important to note that a different experimental set up (aerosol versus contact transmission experiments), including a different inoculum dose, was used for the experiments with the rVN1203-based viruses, relative to the experiments with the rPan99-based viruses. Thus, the results shown in Figure 4 should not be compared directly to those presented in Figure 5. As shown in Figure 5A, the rVN1203 wild-type virus transmitted rapidly to three of four contact guinea pigs. In contrast, the rVN1203 PB2 627E mutant transmitted to only one contact animal (Figure 5B). While this result suggests that this single amino acid change – PB2 K627E – reduces the transmission potential of a highly pathogenic avian influenza virus in a mammalian species (p = 0.09, Student's t-test), an increased number of replicates would be required to prove a statistically significant reduction in transmission. Furthermore, due to the space constraints and high cost of housing animals under BSL3-enhanced containment, we did not retain guinea pigs infected with VN1203 virus for the three weeks required to obtain convalescent sera for HI assays in order to assess transmission to contact animals by seroconversion.

The PB2 gene of the transmitted virus was subjected to RT PCR and sequencing and the introduced mutation, K627E, was found to be maintained. In contrast, virus isolated from three of the inoculated animals late in the course of infection (day 6 p.i.) exhibited large plaque phenotypes, suggesting reversion had occurred. The sequencing of one of these isolates confirmed the presence of lysine at PB2 627. As shown in Figure 5C, the rVN1203 627E 701N double mutant virus spread to all four contact guinea pigs. Although slightly more efficient (100% compared to 75% for the wild-type virus), transmission of the rVN1203 627E 701N double mutant virus appeared to occur with slower kinetics than the wild-type, based on the relatively low titers isolated from contact animals 24 h after the initiation of contact. Taking into account the virus titers shed by the inoculated animals and the 50% infectious doses of the wild-type and double mutant viruses (see below and Table 3), it is unclear why the kinetics of transmission differ. Again, sequencing of the PB2 gene of one transmitted virus indicated that both the K627E and D701N mutations were retained. Thus, the amino acid change PB2 D701N favors the transmission of an H5N1 virus in a mammalian host and this mutation fully compensates for the lack of a lysine at position 627 in terms of transmission by the contact route.

**Table 3:** Guinea pig 50% infectious dose of wild-type and PB2 mutant viruses.

	rWild-type*	PB2 627E*	PB2 627E 701N*
Pan99 background	24	215	22
VN1203 background	3	22	3

*: GPID50 values are shown in units of PFU/guinea pig.

Despite productive growth of all three VN1203-based viruses in the guinea pig respiratory tract, clinical signs were not observed following either intranasal inoculation or infection through contact transmission. The average peak nasal wash titers (reached on day 2 p.i.) of guinea pigs intranasally inoculated with the rVN1203 wild-type and 627E mutant viruses were similar, at approximately 2.3×10^5^ PFU/ml. The titers of the double mutant virus shed by inoculated guinea pigs at day 2 p.i. were higher, at about 1.1×10^6^ PFU/ml. Statistical analysis indicated that only the difference between the wild-type and double mutant virus was significant, with a p value of 0.044 (Student's t-test). Overall, the data do not convincingly show that enhanced shedding from the upper respiratory tract contributes to improved transmission of the rVN1203 wild-type virus. In a separate experiment designed to investigate a possible role in transmission of shedding in the feces, we collected rectal swab samples from guinea pigs inoculated with wild-type rVN1203 virus on days 2, 4, and 6 post-infection. No virus was detected in these rectal swab samples, despite efficient isolation of virus from the upper respiratory tract of the same animals. *50%*

### Infectious dose of rPan99 and rVN1203-based viruses in guinea pigs

In both the rPan99 and rVN1203 backgrounds, the identities of PB2 residues 627 and 701 had a clear effect on transmission. Nevertheless, contrary to our expectations, mutation of these amino acids in the rVN1203 background did not have a striking effect on peak viral growth in the upper respiratory tract of guinea pigs (Figure 5). In the rPan99 background, differences in shedding titers were seen on day 2 p.i., which may have contributed to the transmission phenotypes observed. The effect, however, was limited to day 2 p.i., with similar peak titers being reached by all three viruses by day 4 p.i. (Figure 3A and Figure 4). We therefore reasoned that the reduction in transmissibility may be due to an effect on the recipient, as well as possibly the donor, host in the transmission equation. In other words, we predicted that the 50% infectious dose (ID_50_) of the PB2 627E containing viruses would be higher than either the wild-type or the double mutant viruses. To test this prediction, we inoculated groups of four guinea pigs with 10-fold serial dilutions of each of the six recombinant viruses to determine the GPID_50_ in each case. The results are summarized in Table 3. In both virus backgrounds, the GPID_50_ of the wild-type and 627E 701N double mutant viruses were found to be very similar or the same. In contrast, the rPan99 627E virus had a GPID_50_ of approximately 10-fold higher, and the rVN1203 627E virus had a GPID_50_ of approximately 7-fold higher than the corresponding wild-type viruses. Although the differences are marginal, it is possible that decreased infectivity contributes to the inefficient transmission phenotypes of both 627E-containing viruses.

## Discussion

In the context of an influenza virus which is well adapted to the human host, rPan99, and an avian-like influenza virus isolated from a human host, rVN1203, we have shown that polymorphisms at residues 627 and 701 of PB2 influence transmission among guinea pigs. In addition, we have tested the hypothesis proffered by de Jong et al. [1] that, with respect to adaptation of an avian virus to a mammalian host, D701N can functionally replace E627K. We found that, when PB2 627 is a glutamic acid residue, the D701N mutation not only improves viral growth in mammalian cells, but enhances transmission between guinea pigs.

Using minigenome systems and infected cell cultures, the introduction of the E627K mutation into an avian PB2 gene has previously been shown to increase polymerase activity and viral growth at 33°C [6],[10]. With the mutation of the wild-type K627 to E in the rVN1203 background, we saw a marked reduction in plaque size at both 33°C and 37°C. The degree to which the plaque size decreased relative to the rVN1203 wild-type virus was greater at 33°C, however, as would be expected based on earlier reports. In the rPan99 background, the reduction in plaque size observed with the K627E mutation was again seen at both temperatures and was more subtle than for rVN1203. The observation that the fully human-adapted virus rPan99 was better able to tolerate the PB2 K627E mutation and maintain plaque formation at 33°C than the more avian-like rVN1203 virus may indicate that Pan99 has developed degenerate mechanisms to permit growth at the lower temperature of 33°C.

The *in vivo* growth of the rPan99 627E virus was impaired relative to the wild-type, but only early (day 2) after infection. Although improved growth in the upper respiratory tract early after infection may act to increase the efficiency of transmission, the fact that – at 4 d p.i. – the rPan99 627E and rPan99 627E 701N mutant viruses reach similar peak titers to the wild-type may argue against this as a mechanism for the improved transmission of the wild-type virus. In addition, contrary to recent findings in mice [12], we did not observe marked differences in the viral titers reached by rVN1203 wild-type, rVN1203 627E and rVN1203 627E 701N viruses in the upper respiratory tract of guinea pigs. We did, however, see a clear effect of the PB2 mutations on transmission in the rVN1203 background, as well as in the rPan99 background. We hypothesized that the observed transmission phenotypes might arise from differences in infectivity. Determination of the GPID_50_ values of the three rVN1203- and the three rPan99-based viruses did reveal that higher doses of the 627E mutant viruses are required to productively infect guinea pigs. Although suggestive, the differences observed (approximately 7-fold for rVN1203 and 10-fold for rPan99) may be within the range of error of the GPID_50_ assay. Thus, the mechanism underlying the decreased transmission efficiencies of rVN1203 PB2 627E and rPan99 PB2 627E viruses relative to the corresponding wild-type and double mutant viruses remains uncertain. Our data suggest that reduced viral growth in the nasal passages or decreased infectivity may play a role, but do not allow us to draw firm conclusions on this point. One additional possibility is that, despite reaching similar peak titers in nasal lavage, the 627E mutant viruses are not shed into the air as efficiently as the wild-type and double mutant viruses.

For all three rVN1203 viruses, the viral growth kinetics observed in inoculated animals were markedly different from those in the contact animals. This is not normally observed with human-adapted influenza viruses in guinea pigs [7],[8],[22], but probably reflects a dose-dependent growth phenotype of rVN1203 in this host.

In terms of viral growth *in vitro* and *in vivo* as well as transmission between guinea pigs, the introduction of N at position 701 was found to at least partially reverse the phenotype of the K627E mutant viruses in both virus backgrounds tested. Further support for the idea that 701N can compensate for a lack of 627K in mammalian hosts is found in the published sequences of swine influenza PB2 genes. Among the avian-like H3N2 viruses circulating in swine, European isolates for which sequences are available in Genbank carry 627E with 701N, while Asian isolates predominantly carry 627K with 701D. Although the number of transmission events is low, it may also be of note that three swine influenza viruses isolated from humans in Asia possessed PB2 627E and 701N [23],[24].

In wild birds, H5N1 viruses carrying PB2 627K arose during the Qinghai Lake outbreak in 2005; descendants of these viruses retaining possession of 627K continue to circulate among wild waterfowl [25]. Furthermore, the presence of PB2 627K or 701N has been reported in multiple human isolates of H5N1 influenza viruses [1],[17],[18]. Despite the relatively high incidence of these mammalian-adaptive mutations in human H5N1 isolates, sustained human-to-human spread has not been observed. Thus, although our data show that PB2 627K or 701N is necessary for optimal transmission of either human- or avian-adapted viruses between guinea pigs, these mutations are not sufficient to support human-to-human spread of H5N1 influenza. Indeed, studies in the ferret model indicate that transmissibility is a complex, multigenic trait [2],[5]. The appropriate receptor specificity is most likely required [4],[26]; we have shown that the PB2 protein plays a role; and other viral factors most likely contribute to the transmission phenotype.

We have identified PB2 627K and 701N as determinants of transmission between mammals. The observation that these residues contribute to the transmission of both H3N2 and H5N1 influenza viruses between mammals suggests that adaptations in the PB2 protein may be a prerequisite of transmission common to all influenza A subtypes. The identification of the additional viral factors necessary for—and ultimately the set of traits sufficient to support—human-to-human spread will greatly improve our understanding of how a pandemic strain arises.

## Materials and Methods

### Cells

Madin Darby canine kidney (MDCK) cells were maintained in minimum essential medium (Gibco) supplemented with 10% fetal bovine serum, 100 units/mL of penicillin, and 100 µg/mL of streptomycin. 293T cells were maintained in Dulbecco's minimum essential medium (Gibco) supplemented with 10% fetal bovine serum.

### Rescue of recombinant viruses

Recombinant Pan99 and VN1203 viruses were rescued according to previously reported protocols, with minor modifications [27],[28]. Briefly, 8 pPOL1-based vRNA expression plasmids and 7 pCAGGS-based support plasmids (for the expression of NS1, NA, NP, HA, PA, PB1 and PB2) were used to transfect 293T cells. At 24 h post-transfection, growth medium was replaced with serum-free medium containing 1 µg/mL TPCK-treated trypsin (Sigma) and MDCK cells were added to the culture. At 72 h post-transfection, rescue supernatant was subjected to plaque assay on MDCK cells in order to obtain clonal isolates. Rescued viruses were propagated in 10 d old embryonated hens' eggs (Pan99-based) or MDCK cells (VN1203-based) at 37°C. Genotypes of wild-type viruses were verified by RT-PCR and partial sequencing of each of the eight segments. For the rescue of mutant viruses, site-directed mutagenesis was used to introduce the required mutations into the pPol1Pan99-PB2 or pPol1VN1203-PB2 plasmids using the QuikChange II Site Directed Mutagenesis kit (Stratagene). Rescue protocols were as for the wild-type viruses. Genotypes of the mutant viruses were confirmed through RT-PCR and sequencing of the respective PB2 segments. The presence of a multibasic cleavage site in the HA of rVN1203 wild-type and mutant viruses was confirmed by verifying their ability to form plaques in the absence of trypsin (data not shown).

### Viral growth in MDCK cells

Characterization of plaque phenotypes on MDCK cells was performed as described previously [29]. All work with VN1203-based viruses was performed in a USDA and CDC-approved biosafety level 3+ containment laboratory in accordance with institutional biosafety requirements. Polyclonal rabbit anti-A/Puerto Rico/8/34 was used as the primary antibody, and Amersham ECL™-HRP linked anti-rabbit was used as the secondary antibody for immunostaining.

To examine multi-step growth, MDCK cells were infected at a multiplicity of 0.01 PFU/cell. Following a 45 min incubation, inoculum was removed and monolayers were washed with PBS. Dishes were then incubated at 33°C or 37°C, as indicated, and samples of growth medium collected at 3, 10, 24, 48 and 72 h p.i (for rPan99-based viruses) or at 2, 24, 48, and 72 h.p.i. (for rVN1203-based viruses). Titers were determined by plaque assay on MDCK cells.

### Animals

Female Hartley strain guinea pigs weighing 300–350 g were obtained from Charles River Laboratories Inc. (Wilmington, MA). Animals were allowed free access to food and water and kept on a 12 h light/dark cycle. Guinea pigs were anesthetized for the collection of blood and of nasal wash samples, using a mixture of ketamine (30 mg/kg) and xylazine (2 mg/kg), administered intramuscularly. All procedures were performed in accordance with the Institutional Animal Care and Use Committee guidelines. During guinea pig transmission experiments strict measures were followed to prevent aberrant cross-contamination between cages: exposed animals were handled before inoculated animals, gloves were changed between cages, and work surfaces were sanitized between guinea pigs.

### Viral growth in guinea pig respiratory tract

Three groups of four guinea pigs were inoculated with 1000 PFU in a 300 µl volume intranasally: group 1 received rPan99, group 2 received rPan99 PB2 627E, and group 3 received rPan99 PB2 627E 701N. At 2 d p.i., nasal washings were collected from all 12 guinea pigs. Six guinea pigs were then sacrificed for the collection of lung tissue. At 4 d p.i., nasal washes were collected from the remaining 6 animals; these 6 guinea pigs were then sacrificed and the lungs removed. As described previously [22], nasal washing was performed by instilling 1 mL PBS-BA-PS (PBS supplemented with 0.3% bovine serum albumin, 100 units/mL of penicillin, and 100 µg/mL of streptomycin) into the nostrils and allowing it to drain onto a Petri dish. Samples were collected into 1.5 ml tubes and centrifuged for 5 min at 2000×g and 4°C; supernatants were stored at −80°C prior to analysis by plaque assay. For the collection of lung tissue, animals were killed through exposure to CO_2_ gas. After the lungs were removed, three lobes were detached, placed in separate Petri dishes, and minced. Approximately 300 mg of each lobe was then transferred to 4 ml cold PBS-BA-PS. Thus, three samples were taken from each animal. Extracted lung tissue was weighed, homogenized in PBS-BA-PS and centrifuged at 12 000×g and 4°C for 10 min to pellet debris. Immediately after preparation, supernatants were serially diluted and subjected to plaque assay.

Data in Figure 3A represent the nasal wash titers on days 2 and 4 post-infection for all animals inoculated intranasally with the indicated viruses. This data includes titers derived from inoculated animals used in aerosol transmission experiments as well as the 12 inoculated animals used to assess viral growth in the upper and lower respiratory tracts.

### Aerosol transmission experiments

As in our previous publications, the term “aerosol” is used herein to describe respiratory droplets of all sizes.

Aerosol transmission experiments—performed with rPan99 and Pan99-derived mutants—were carried out as described previously [7]. Each experiment involved eight guinea pigs; the data shown in Figure 4 represent cumulative results from two independent experiments for rPan99 wild-type, and three independent experiments for the two rPan99 PB2 mutant viruses. Briefly, the procedure was as follows. On day zero, four of the eight guinea pigs were inoculated intranasally with 1000 PFU of virus. At 24 h p.i., each of the eight guinea pigs was transferred to a “transmission cage” and then placed into an environmental chamber (Caron model 6030) set to 20% relative humidity and 20°C. Each infected animal was paired on a shelf with a naïve animal; in this arrangement, no contact between the guinea pigs is possible. The animals were housed in this way for seven days, after which they were removed from the chamber and separated. Nasal wash samples were collected from anesthetized guinea pigs on days 2, 4, 6, 8, and 10 p.i. by instilling 1 mL of PBS-BA-PS into the nostrils and collecting the wash in a Petri dish. Samples were stored at −80°C prior to analysis. Titers in nasal wash samples were determined by plaque assay of 10-fold serial dilutions on MDCK cells.

### Contact transmission experiments

Contact transmission experiments were performed with rVN1203 wild-type and rVN1203-derived PB2 mutant viruses. This work was carried out in a USDA and CDC-approved biosafety level 3+ containment laboratory in accordance with institutional biosafety requirements. For each virus, four of eight guinea pigs were inoculated intranasally with 10^4^ PFU. At 24 h p.i., each infected animal was placed in the same cage with one naïve animal, and animals were co-housed in this way for a total of seven days. Nasal washes were collected from all guinea pigs on days 2, 4, 6 and 8 p.i. Samples were stored at −80°C prior to analysis by plaque assay. Animals were euthanized through intraperitoneal injection of 100 mg/kg sodium pentobarbital (Nembutal; Ovation Pharmaceuticals, Inc.) on day 8 p.i. These experiments were performed under ambient conditions (19–22°C and 30–70% relative humidity).

### Determination of guinea pig 50% infectious dose

Groups of four guinea pigs were inoculated intranasally with ten-fold serial dilutions of each virus stock. At 2 d.p.i. nasal washings were collected from all guinea pigs. The nasal wash samples were analyzed by plaque assay on MDCK cells to determine which guinea pigs were infected. The GPID_50_ was then calculated by the method of Reed and Muench [30] and expressed in PFU per guinea pig.

### Accession numbers

PB2 segment of A/Panama/2007/99: Genbank accession number DQ487334.

PB2 segment of A/Viet Nam/1203/04: Genbank accession number AY651719.
